# Not Guilty

**Published:** 1870-04

**Authors:** W. E. Driscoll

**Affiliations:** Bedford, Ind.


### NOT GUILTY.

BY W. E. DRISCOLL, BEDFORD, IND.

The article on "Safety from Exploding Vulcanizers" in the March number of the REGISTER, places me in a false position. Since writing the article, last November, I have *entirely* abandoned rubber; and feel humiliated and disgraced to think that those reading my communication will think I am so "weak or wicked" as to have anything further to do with it. Instead of in any way encouraging its use, I wish I could thunder it, like the trump of resurrection, into the ears of all who are its slaves, to rise and shake off its whole train of degrading influences. Touch not, taste not, handle not the unclean thing, is the only course consistent with honor, with patients or respectability in the profession.

Does any one ask what we are to do for a *cheap* substitute? Any of the metals or alloys that have so far been advertised for the purpose are incomparably better, even for the shortest gums in full upper cases. I suppose the main reason for this is because of the superior heat conducting quality of the metal over rubber, producing less irritation with a sharply defined air chamber. But there are other reasons obvious enough to any one qualified to judge.

